# Regulation of d-Aspartate Oxidase Gene Expression by Pyruvate Metabolism in the Yeast *Cryptococcus humicola*

**DOI:** 10.3390/microorganisms9122444

**Published:** 2021-11-27

**Authors:** Daiki Imanishi, Sota Zaitsu, Shouji Takahashi

**Affiliations:** Department of Bioengineering, Nagaoka University of Technology, Nagaoka, Niigata 940-2188, Japan; s175037@stn.nagaokaut.ac.jp (D.I.); s193321@stn.nagaokaut.ac.jp (S.Z.)

**Keywords:** d-aspartate oxidase, pyruvate carboxylase, *Cryptococcus humicola*, d-aspartate, gene expression

## Abstract

d-Aspartate oxidase (DDO) is a peroxisomal flavoenzyme that catalyzes the oxidative deamination of acidic d-amino acids. In the yeast *Cryptococcus humicola* strain UJ1, the enzyme ChDDO is essential for d-Asp utilization and is expressed only in the presence of d-Asp. Pyruvate carboxylase (Pyc) catalyzes the conversion of pyruvate to oxaloacetate and is involved in the import and activation of certain peroxisomal flavoenzymes in yeasts. In this study, we analyzed the role of Pyc in the expression of *ChDDO* gene in *C. humicola* strain UJ1. *PYC* gene disruption (∆*Chpyc1*) in strain UJ1 resulted in growth retardation on glucose and NH_4_Cl medium. The growth was restored by supplying oxaloacetate from l-Asp or α-ketoglutarate by a transaminase. On the other hand, the supply of oxaloacetate from d-Asp by ChDDO was not able to prevent growth retardation because of a significant decrease in *ChDDO* gene expression at the transcriptional level. The addition of pyruvate significantly decreased *ChDDO* gene transcription in the ∆*Chpyc1* strain but increased the same in the wild-type strain, even though the intracellular pyruvate content was similar in both strains. These results suggest that *ChDDO* gene expression might be regulated by pyruvate metabolism, as well as by the presence of d-Asp.

## 1. Introduction

d-Aspartate oxidase (DDO, EC 1.4.3.1) catalyzes the oxidative deamination of acidic d-amino acids to yield corresponding α-keto acids and ammonia. The same reaction for neutral and basic d-amino acids is catalyzed by d-amino acid oxidase (DAO, EC 1.4.3.3). Both enzymes are localized in peroxisomes and require flavin adenine dinucleotide (FAD) as a cofactor for enzyme activity. Due to their common catalytic mechanism and sequence similarity, they are believed to have derived from a common precursor sequence [1,2]. DDO has been discovered in various eukaryotic organisms, ranging from fungi to mammals but so far, it has not been found in plants or prokaryotic organisms [3,4,5,6,7]. In fungi, DDO participates in the assimilation and detoxification of acidic d-amino acids [8]. In nematodes, three DDOs participate in fertility and hatching, and one of them might also be involved in the regulation of growth and longevity [9,10,11]. In mammals, DDO is responsible for the degradation of endogenous and exogenous d-Asp and the regulation of d-Asp levels in several tissues [12,13]. Thus, it plays an important role in various physiological processes such as hormone secretion and neurotransmission [12,13]. Recently, it has also been reported that DDO function is associated with the psychiatric disorder schizophrenia in humans [14,15,16,17]. Therefore, to expound on the physiological significance of DDO in eukaryotes, it is important to understand the environmental and protein factors that can affect the expression of DDO.

The yeast *Cryptococcus* (*Vanrija*) *humicola* strain UJ1 can grow on d-Asp as the sole source of carbon, nitrogen, or both because of the action of DDO (ChDDO) [7,8]. *ChDDO* gene expression is specifically induced only by the presence of d-Asp in the culture medium at the transcriptional level [8]. Such acidic d-amino acid-dependent induction of DDO activity has also been observed in other organisms. In the fungus *Fusarium sacchari* var. *elongatum* strain Y-105 and the yeast *Candida boidinii* strain 2201, DDO activity increases when grown on acidic d-amino acids as the sole source of nitrogen [18,19]. In mice, treated orally or intraperitoneally with d-Asp, DDO activity increases in the liver and kidneys [20]. Oral administration of d-Asp to pregnant rats increases DDO activity in the liver and kidneys of newborn rats [12]. These findings suggest that the mechanism of *DDO* gene expression induced by acidic d-amino acids might be distributed in eukaryotic organisms. However, the precise regulatory mechanism underlying the expression remains undetermined.

Pyruvate carboxylase (Pyc, EC 6.4.1.1) is widely distributed in bacteria, fungi, plants, and animals, and functions as an anaplerotic enzyme that supplies oxaloacetate to the TCA cycle [21,22]. In the methylotrophic yeasts *Hansenula* (*Ogataea*) *polymorpha* and *Pichia* (*Komagaella*) *pastoris*, Pyc1 can activate alcohol oxidase (AO, EC 1.1.3.13) [23]. In methylotrophic yeast strains lacking Pyc1, newly synthesized AO monomers cannot bind to FAD, preventing their transport to the peroxisome and the formation of enzymatically active octamers [23]. Since the enzymatic activity of Pyc1 is not involved in AO assembly and the protein can interact with the AO protein, Pyc1 is believed to have a moonlighting function in FAD-binding to AO monomers in yeasts [23,24,25]. The moonlighting function helps avoid the enzymatic generation of H_2_O_2_ and formaldehyde in the cytoplasm for utilizing large amounts of alcohol [23,26]. Recently, it has been reported that *PYC1-*gene deletion in *P. pastoris* results in a growth defect when grown on d-Ala medium and a reduction in DAO enzyme activity, suggesting that Pyc1 could also be involved in DAO activation [27]. Since DAO and AO are peroxisomal enzymes with FAD as a cofactor, DAO is considered to be activated by Pyc1 via the same mechanism as proposed for AO. However, it is unknown whether Pyc is involved in DDO activation.

In this study, we investigated the role of Pyc in the inducible expression of *ChDDO* gene by d-Asp in *C. humicola* strain UJ1. For this investigation, we identified the *PYC1* gene (*ChPYC1*) of *C. humicola* UJ1 and created a *ChPYC1*-disrupted strain. In the gene-disrupted strain, the induction level of DDO activity and *ChDDO* gene transcription was significantly decreased. The results of this study suggest that the expression of *ChDDO* gene might be regulated at the transcriptional level, not only by d-Asp-specific induction, but also by pyruvate metabolism.

## 2. Materials and Methods

### 2.1. Materials

Yeast nitrogen base without amino acids (YNB) and YNB without amino acids and ammonium sulfate (YNB w/o AA and AS) were obtained from Difco (Detroit, MI, USA). d-Asp was a generous gift from Mitsubishi Tanabe Pharmaceutical (Osaka, Japan). All other chemicals were purchased from Wako Pure Chemical Industries (Osaka, Japan), Nacalai Tesque (Kyoto, Japan) or Sigma-Aldrich (St Louis, MO, USA). DNA polymerase was purchased from Takara Bio (Shiga, Japan). PCR primers (Table S1) were synthesized by Eurofins Genomics (Tokyo, Japan).

### 2.2. Strains, Media, and Growth Conditions

The *C. humicola* strain UJ1 [7] was used as the wild-type strain. *C. humicola* strain UM3 [28], a *ura3* gene mutant derived from strain UJ1, was used as the host for gene disruption experiments. *C. humicola* strain Δ*Chdd*o [8], a *ChDDO* gene-disrupted strain, was used for intracellular d-Asp quantification. *Saccharomyces cerevisiae* strain W303-1A (*MAT*a *leu*2-3/112, *ura*3-1, *trp*1-1, *his*3-11/15, *ade*2-1, *can*1-100) was used as the host for *ChPYC*1 gene expression [29]. Yeast cells were routinely grown in YPD medium (1% (*w*/*v*) yeast extract, 2% (*w*/*v*) peptone, 2% (*w*/*v*) glucose), or in SD medium (0.67% (*w*/*v*) YNB, 2% (*w*/*v*) glucose). Where required, 20 μg/mL uracil, 20 μg/mL adenine, 20 μg/mL l-Trp, 100 μg/mL l-Leu, 20 μg/mL l-His, and 10 mM l-Asp were added to SD medium. The growth test of the *ChPYC*1 gene-disrupted strain (*∆Chpyc*1) was performed as follows. The cells were precultured in SD medium at 30 °C for 16 h. An aliquot of the preculture was inoculated into a synthetic medium containing 0.56% (*w*/*v*) YNB w/o AA and AS, the carbon, and the nitrogen sources such that cell density of OD_600_ = 0.01. The initial pH was adjusted to 7.0 or 4.0. For solid medium, 2% (*w*/*v*) agar was added to the culture medium. *Escherichia coli* strain DH5α was used as a host for plasmid propagation. *E. coli* cells were grown at 37 °C in LB medium (1% (*w*/*v*) tryptone, 0.5% (*w*/*v*) yeast extract, 0.5% (*w*/*v*) NaCl) supplemented with 100 μg/mL ampicillin.

### 2.3. DNA and RNA Preparation

The total DNA of *C. humicola* was prepared as previously described [28]. For the preparation of total RNA, cells were washed twice with ice-cold water, resuspended in ice-cold water, and then transferred to a 2 mL screw tube containing an equal volume of φ0.45–0.5 mm zirconia beads (BioSpec Products, Inc., Bartlesville, OK, USA). After centrifugation at 12,000× *g* for 2 min at 4 °C, the supernatant was removed, and the cells were lyophilized using a freeze dryer system (DRC-1100 and FDU-2100, EYELA, Tokyo, Japan) and stored at −80 °C until use. The tube containing the lyophilized cells was shaken vigorously for 5 min using a vortex mixer. Total RNA was extracted and purified from the disrupted cells using a Direct-zol RNA MiniPrep Kit (ZYMO Research, Irvine, CA, USA) according to the manufacturer’s instructions.

### 2.4. Cloning and Expression of ChPYC1 Gene

The putative Pyc1-encoding gene (*ChPYC*1) of strain UJ1 was identified in the yeast draft genome sequence data [30] via a BlastP search [31] using the amino acid sequences of *P. pastoris* Pyc1 (Uniprot: P78992). The cDNA of the *ChPYC*1 gene was synthesized by reverse transcription PCR using total RNA as template, SuperScript IV One-Step RT-PCR System (Invitrogen, Thermo Fisher Scientific, Waltham, MA, USA), and the primer set ChPyc1F1/ChPyc1R1 (Table S1). To construct a *ChPYC*1 gene expression vector in *S. cerevisiae*, the entire ORF of the *ChPYC1* gene was amplified by PCR using KOD FX Neo DNA polymerase (Toyobo, Osaka, Japan) with the primer set pWGP3ChPyc1F/pWGP3ChPyc1R using the cDNA as a template. The linearized pWGP3 was generated by PCR using the yeast plasmid vector as a template with the primer set pWGP3R/pWGP3F [32] and ligated with the *ChPYC1* gene using the In-Fusion HD Cloning Kit (Takara Bio) to obtain the expression plasmid pWGP3_*ChPYC*1, where the *ChPYC*1 gene was expressed under the control of the glyceraldehyde 3-phosphate dehydrogenase promoter.

### 2.5. Pyc Activity Assay

The cells were precultured in SD medium at 30 °C for 16 h. An aliquot of the preculture was transferred to a fresh SD medium at a final cell density of OD_600_ = 0.05. The cells were further grown at 30 °C for 16 h with shaking at 166 rpm. The cells were washed twice with ice-cold lysis buffer (100 mM Tris-HCl, pH 8.0), resuspended in the same buffer, and then transferred to a 2 mL screw tube containing an equal volume of φ0.45–0.5 mm glass beads. The tube was shaken vigorously for 1 min using a Mini Bead Beater-8 (BioSpec Products, Bartlesville, OK, USA), followed by cooling on ice for 3 min. This procedure was repeated eight times. The extract was clarified by centrifugation at 20,000× *g* for 30 min at 4 °C, and the supernatant was used as the crude cell extract for the enzyme assay. The activity was assayed spectrophotometrically using the malate dehydrogenase (MDH) coupled assay method described by Payne and Morris [33]. Oxaloacetate produced by Pyc was converted to malate by MDH. Simultaneously, the disappearance of NADH was observed at 340 nm. The activity was calculated using a molar extinction coefficient of 6.22 mM^−1^·cm^−1^. The reaction mixture contained 7 mM pyruvate, 15 mM KHCO_3_, 5 mM Mg_2_Cl, 1 mM ATP, 0.005 mM acetyl-CoA, 0.23 mM NADH, and 1 U/mL MDH in 100 mM triethanolamine buffer (pH 8.0).

### 2.6. Disruption of ChPYC1 Gene

A *ChURA3* fragment, approximately 2.0 kbp long, was amplified using Tks Gflex DNA polymerase (Takara Bio) with the primer set URA3F/URA3R (Table S1) and the yeast vector pICUG as a template [28]. Approximately 1.0 kbp of the 5′- and 3′-regions of *ChPYC1* were amplified using the following primer sets: ChPYC1UF/ChPYC1UR for the *ChPYC1* 5′-region and ChPYC1DF/ChPYC1DR for the *ChPYC1* 3′-region (Table S1). Yeast genomic DNA was used as the template. The amplified *ChURA3* and 5′- and 3′-fragments of *ChPYC1* were mixed, and the fused DNA fragments were obtained via overlap extension PCR using the 5′- and 3′-terminal primers for the *ChPYC1* gene. The resulting disruption cassette for the *ChPYC1* gene (approximately 5.0 kbp) was introduced into the yeast cells via electroporation using a MicroPulser Electroporator (Bio-Rad, Hercules, CA, USA) as described previously [28]. Transformant was selected for their ability to grow on SD medium without uracil at 30 °C for 3 days. The disruption of *ChPYC1* was confirmed by PCR using the primer sets Fwd1/Rev1 and Fwd2/Rev2 for *ChPYC1* (Table S1). In the gene disruption mutant, *ChURA3* was inserted into the genomic *ChPYC1* in the opposite direction between 977 and 1749 bp.

### 2.7. ChDDO Gene Induction Experiment

The cells were precultured in SD medium at 30 °C for 16 h. An aliquot of the preculture was transferred to a fresh SD medium at a final cell density of OD_600_ = 0.05, and the cells were further grown at 30 °C for 16 h with shaking at 166 rpm. The cells were collected by centrifugation and washed twice with ice-cold water, and 50 OD_600_ units of cells were resuspended in a synthetic medium (YNB w/o AA and AS) containing 60 mM d-Asp and 60 mM pyruvate. After incubation at 30 °C for 5 h with shaking, the cells were harvested by centrifugation and washed twice with ice-cold water.

### 2.8. DDO Activity Assay

The DDO activity was measured as described by Takahashi et al. [8]. Describing briefly, cells were washed twice with ice-cold lysis buffer (50 mM potassium phosphate, pH 8.0, 2 mM EDTA), resuspended in the same buffer, and then transferred to a 2 mL screw tube containing an equal volume of φ0.45–0.5 mm glass beads. The tube was shaken vigorously for 1 min using a Mini Bead Beater-8 (BioSpec Products), followed by cooling on ice for 3 min. This procedure was repeated eight times. The extract was clarified by centrifugation at 20,000× *g* for 30 min at 4 °C, and the supernatant was used as the crude cell extract for the enzyme assay. Enzymatic activity was determined spectrophotometrically using an HRP-coupled assay with phenol and 4-aminoantipyrine (4-AA) [34]. The reaction mixture contained 20 mM d-Asp, 20 µM FAD, 2 mM phenol, 1.5 mM 4-AA, and 2.5 U/mL HRP (Sigma-Aldrich) in 50 mM potassium phosphate buffer (pH 8.0). The activity was determined at 505 nm using a molar extinction coefficient for the generated quinone imine of 6580 M^−1^·cm^−1^.

### 2.9. Intracellular d-Asp and Pyruvate Quantification

The cells induced as described above were washed twice with ice-cold water, resuspended in ice-cold water, and then transferred to a 2 mL screw tube containing an equal volume of φ0.45–0.5 mm glass beads. After centrifugation at 12,000× *g* for 2 min at 4 °C, the supernatant was removed, and the cells were lyophilized using a freeze dryer system (DRC-1100 and FDU-2100, EYELA, Tokyo, Japan) and stored at −80 °C until use. The cells were resuspended in ethanol, and the tube was shaken vigorously for 5 min using a Mini Bead Beater-8 (BioSpec Products). The extract was clarified by centrifugation at 20,000× *g* for 30 min at 4 °C, and the supernatant was collected. The supernatant was vacuum-dried at 40 °C and used for d-Asp or pyruvate quantification. Intracellular d-Asp was quantified using the HPLC system described by Kajitani et al. [35]. Intracellular pyruvate was quantified fluorometrically using pyruvate oxidase, HRP, and Amplex UltraRed (Invitrogen, Thermo Fisher Scientific, Waltham, MA, USA), as described by Zhu et al. [36]. H_2_O_2_ produced by pyruvate oxidase reacts with Amplex UltraRed to produce resorufin. The fluorescence intensity of resorufin was measured at excitation/emission wavelengths of 530/590 nm. Pyruvate in the samples was calculated by referring to the calibration curve using standard pyruvate.

### 2.10. Quantitative Real-Time RT-PCR (qRT-PCR)

Total RNA was isolated from cells grown in synthetic medium (YNB w/o AA and AS) containing 60 mM d-Asp. qRT-PCR of *ChDDO* gene transcripts was performed using RNA-direct SYBR Green Realtime PCR Master Mix (Toyobo) with the primer set RTChDDOF2/RTChDDOR2 (Table S1) in a StepOne real-time PCR system (Applied Biosystems, Foster City, CA, USA). Transcription of *TAF10* was determined as a normalizing gene using the primer set RTChTAF10F/RTChTAF10R, and the relative transcriptional levels of the *ChDDO* gene were calculated against the normalizing gene using the 2^−ΔΔC^_T_ method [37].

### 2.11. Sequence Analyses

Amino acid sequence alignment was performed using T-coffee (http://tcoffee.crg.cat/, accessed on 1 December 2020). Sequence comparisons were performed using the EMBOSS needle pairwise sequence alignment (https://www.ebi.ac.uk/Tools/psa/emboss_needle/, accessed on 1 December 2020).

## 3. Results

### 3.1. Identification of Pyc Homolog of C. humicola Strain UJ1

**Figure 1:**
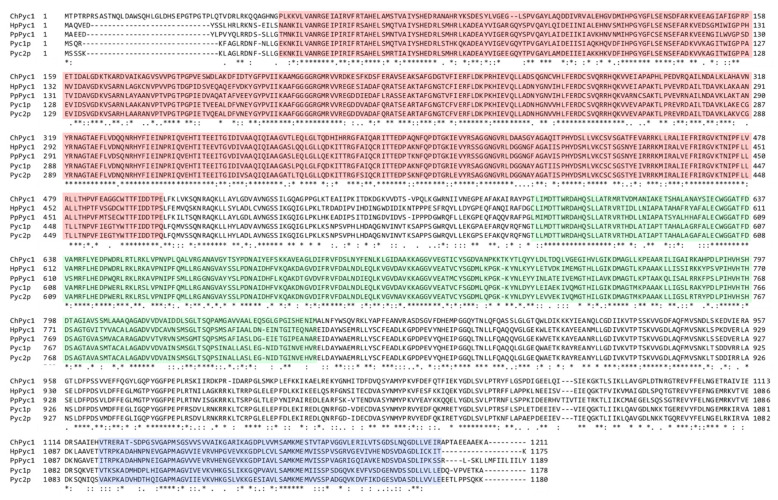
Comparison of amino acid sequences of ChPyc1 with HpPyc1 (*H. polymorpha*, Uniprot: A0A1B7SK73), PpPyc1 (*P. pastoris*, P78992), Pyc1p, and Pyc2p (*S. cerevisiae*, P11154 and P32327, respectively). Identical residues and amino acid substitutions with low and high similarity are indicated by asterisks, dots, and double dots, respectively. The regions boxed in red, blue, and green correspond to BC, TC, and BCC domains, respectively, of Pyc functional domains [38].

To identify a Pyc homolog in *C. humicola* strain UJ1, we searched for a homolog in the draft genome sequence of strain UJ1 using the amino acid sequence of Pyc1 (PpPyc1, Uniprot: P78992) of the yeast *P. pastoris*. We found a sequence displaying significant homology to PpPyc1 and named it ChPyc1. ChPyc1 was 1211 amino acid residues in length, with a calculated molecular mass of approximately 133 kDa. It showed 58.4%, 60.5%, 59.5%, and 59.1% amino acid sequence identity with those of PpPyc1, *H. polymorpha* Pyc1 (HpPyc1), *S. cerevisiae* Pyc1p, and Pyc2p, respectively. Three conserved functional domains of Pyc namely N-terminal biotin carboxylation (BC) domain, central transcarboxylation (TC) domain, and C-terminal biotin carboxyl carrier (BCC) domain were found in ChPyc1 (Figure 1) [38], suggesting that ChPyc1 might function as Pyc in strain UJ1. Pyc usually contains a mitochondrial targeting signal that localizes to the mitochondria in most eukaryotic organisms. In contrast, the enzyme of the yeast *S. cerevisiae*, and the filamentous fungi *Aspergillus nidulans*, *A. terreus*, and *Rhizopus oryzae* lacks a mitochondrial targeting signal and localizes in the cytosol [39]. In the methylotrophic yeasts *H. polymorpha* and *P. pastoris*, Pyc is also found in the cytosol [23]. The absence of mitochondrial targeting signal in ChPyc1 suggested its cytosolic localization.

### 3.2. Expression of ChPYC1 Gene in S. cerevisiae

**Figure 2:**
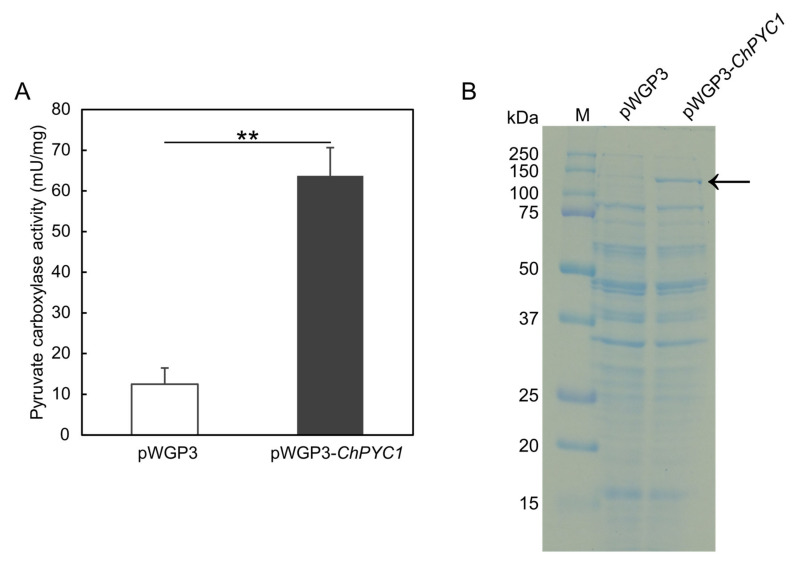
The expression of *ChPYC1* in *S. cerevisiae*. (**A**) Pyruvate carboxylase activity of crude extracts from *S. cerevisiae* cells with (pWGP3-*ChPYC1*) and without *ChPYC1* gene (pWGP3). The activity was assayed spectrophotometrically by malate dehydrogenase (MDH) coupled assay as reported by Payne and Morris [33]. Statistical differences were ascertained by Welch’s *t*-test, ** *p* < 5 × 10^−^^3^. The values are the means of three independent experiments, and the error bars represent the standard deviations. (**B**) SDS-PAGE analysis of the crude extracts from *S. cerevisiae* cells with (pWGP3-*ChPYC1*) and without *ChPYC1* gene (pWGP3). The 10 µg proteins were separated by 12% SDS-PAGE and stained with Coomassie Brilliant Blue. The arrow indicates the protein band corresponding to ChPyc1 (molecular mass: approximately 133 kDa).

To confirm that the *ChPYC1* gene encodes functional Pyc, we constructed an expression plasmid vector, pWGP3_*ChPYC1*, containing a cDNA of *ChPYC1* in the yeast *S. cerevisiae*, where the gene was expressed under the control of glyceraldehyde 3-phosphate dehydrogenase promoter of *S. cerevisiae*. The crude extract from the cells harboring pWGP3_*ChPYC1* exhibited a high Pyc activity (approximately 60 mU/mg protein), whereas that of the cells harboring the control vector (pWGP3) showed a significantly lower activity (approximately 10 mU/mg protein, *p* < 5 × 10^−3^) (Figure 2A). The *ChPYC1* gene expression product in the crude extract was analyzed by performing SDS-PAGE. In the crude extract from the cells expressing the *ChPYC1* gene, a protein band was observed at the position corresponding to the predicted molecular mass of ChPyc1 (approximately 133 kDa), whereas in the control strain not containing the *ChPYC1* gene, the protein band was not observed (Figure 2B). These results indicate that the *ChPYC1* gene encodes a functional Pyc.

### 3.3. Growth Characteristics of ΔChpyc1 Strain

**Figure 3:**
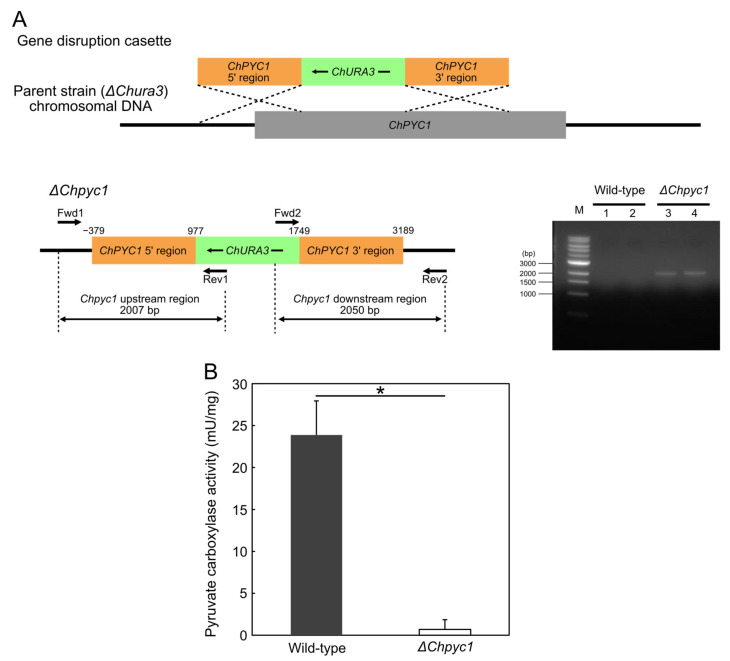
Construction of Δ*Chpyc1* strain, and pyruvate carboxylase activity of the crude extract from the wild-type (strain UJ1) and Δ*Chpyc1* strains of *C. humicola*. (**A**) Schematic representation of *ChPYC1* gene disruption by homologous recombination using *ChURA3* gene and PCR analysis of the gene disruption. The numbers above the box indicate the distance (bp) from the start codon ATG of the chromosomal *ChPYC1* gene. *ChURA3* gene was inserted into the *ChPYC1* gene between 977 and 1749 bp in the opposite direction. Lanes 1 and 2, the negative control amplification of the upstream and the downstream regions, respectively, in the wild-type strain; lanes 3 and 4, the amplification of the upstream and downstream regions in Δ*Chpyc1* strain. PCR was performed using Δ*Chpyc1*-specific primer pairs. (**B**) The activity was measured by the same method as described in Figure 3. Statistical differences were ascertained by Welch’s *t*-test, * *p* < 5 × 10^−^^2^. The values are the means of three independent experiments, and the error bars represent the standard deviations.

**Figure 4:**
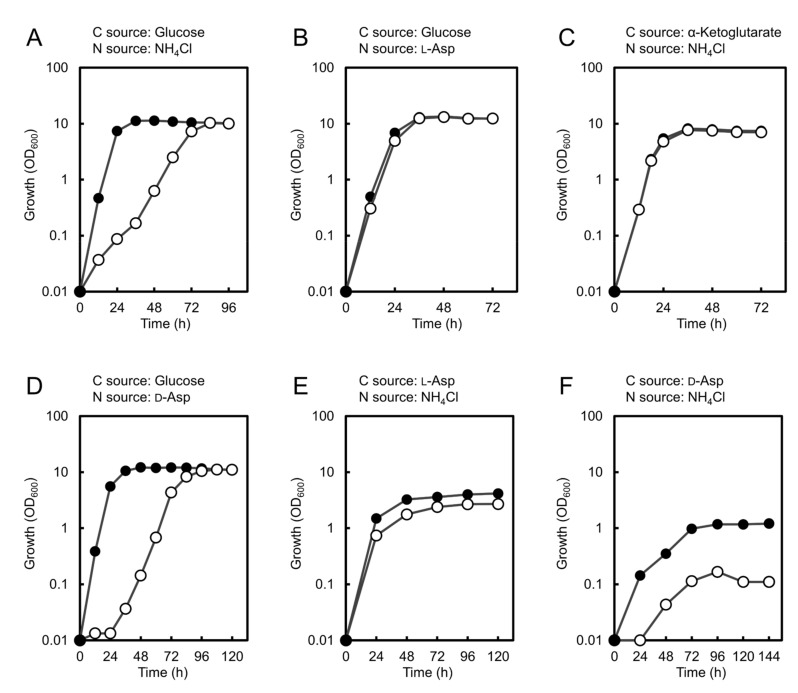
Growth of the wild-type and Δ*Chpyc1* strains on media containing different carbon and nitrogen sources. The wild-type (strain UJ1, closed circle) and Δ*Chpyc1* (open circle) strains were grown in YNB w/o AA and AS media containing either 10 mM NH_4_Cl, l-Asp, or d-Asp as the sole nitrogen source with either 55.5 mM glucose or 66.6 mM α-ketoglutarate as carbon source (**A**–**D**); and in media containing 10 mM ammonium chloride as nitrogen source with either 83.3 mM l-Asp or d-Asp as carbon source (**E**,**F**).

To clarify whether ChPyc1, like Pyc in other organisms, plays a role as an anaplerotic enzyme in the TCA cycle, we constructed a *ChPYC1*-gene disrupted strain (Δ*Chpyc1*) (Figure 3A). The loss of Pyc enzyme function in the Δ*Chpyc1* strain was confirmed by the significant decrease in Pyc activity in the Δ*Chpyc1* strain (*p* < 5 × 10^−2^) (Figure 3B). Next, we analyzed the growth of the Δ*Chpyc1* strain in a medium containing glucose as the sole carbon source and either NH_4_Cl or l-Asp as the sole nitrogen source (Figure 4A,B). The Δ*Chpyc1* strain grew more slowly than the wild-type strain when NH_4_Cl was the sole source of nitrogen (Figure 4A). The growth of Δ*Chpyc1* was recovered when l-Asp replaced NH_4_Cl as the nitrogen source in the medium (Figure 4B). In addition, the growth of Δ*Chpyc1* was also recovered in a medium with α-ketoglutarate as the sole carbon source and NH_4_Cl as the sole nitrogen source (Figure 4C). These results suggest that ChPyc1 plays a role as an anaplerotic enzyme and that the enzymatic function of ChPyc1 can be compensated by supplying oxaloacetate to the TCA cycle by the transaminase reaction between l-Asp and α-ketoglutarate.

**Figure 5:**
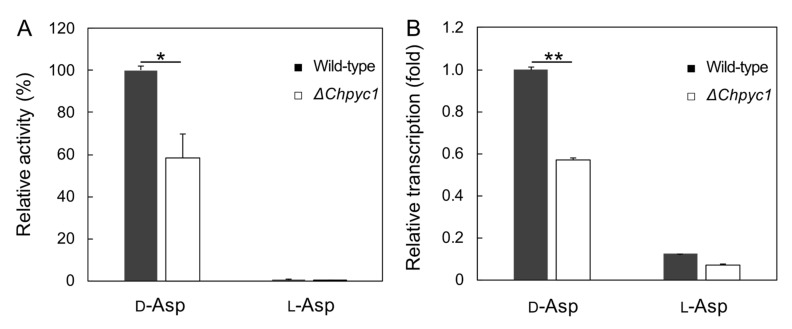
The expression of *ChDDO* gene in the wild-type (strain UJ1) and Δ*Chpyc1* strains. (**A**) DDO activity in the crude extracts from the cells grown in YNB w/o AA and AS medium containing either 60 mM d-Asp or l-Asp as the sole nitrogen and carbon sources. The enzyme activity is expressed as a percentage of that of the wild-type strain on d-Asp. (**B**) Transcription of *ChDDO* gene. The gene transcription was analyzed by qRT-PCR using total RNA from cells grown under the same conditions as in the analysis of DDO activity, normalized to *TAF10* gene transcription, and expressed as a relative ratio of that of the wild-type strain on d-Asp. Statistical differences were ascertained by Welch’s *t*-test, * *p* < 5 × 10^−^^2^; ** *p* < 5 × 10^−3^. The values are the means of three independent experiments, and the error bars represent the standard deviations.

Since d-Asp can be converted to oxaloacetate by ChDDO in strain UJ1, it was theorized that d-Asp could also support the growth of the Δ*Chpyc1* strain. However, d-Asp was not able to recover the growth of the Δ*Chpyc1* strain (Figure 4D,F). This indicates that the reaction of ChDDO with d-Asp cannot compensate for the lack of ChPyc1. Since the lack of Pyc1 in *P. pastoris* causes a decrease in DAO activity [27], it was thought to be due to the decrease in DDO activity in the Δ*Chpyc1* strain. Thus, we analyzed DDO activity in the Δ*Chpyc1* strain. As expected, the enzyme activity in the crude extract of the Δ*Chpyc1* strain induced by d-Asp was approximately 40% lower than that of the wild-type strain (Figure 5A). To further investigate the effect of *ChPYC1* gene disruption on *ChDDO* gene expression, we analyzed *ChDDO* gene transcription. Interestingly, the transcriptional level of the *ChDDO* gene in the Δ*Chpyc1* strain induced by d-Asp was also lower than that in the wild-type strain (Figure 5B). This suggests that *ChPYC1* gene disruption decreased *ChDDO* gene expression at the transcriptional level but not at the post-transcriptional level.

### 3.4. Effect of Pyruvate on the Induction of ChDDO Gene by d-Asp

**Figure 6:**
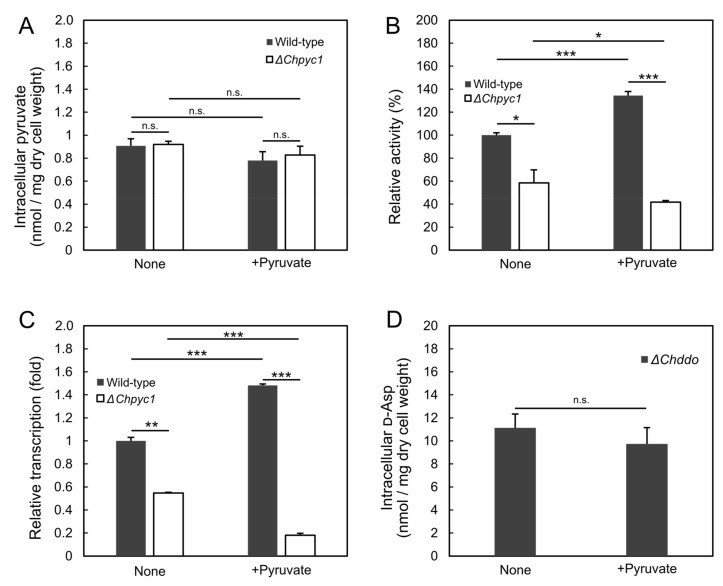
The analysis of the effect of pyruvate on *ChDDO* expression. (**A**) Intracellular pyruvate content in the crude extracts from the cells grown in YNB w/o AA and AS medium containing 60 mM d-Asp with 60 mM pyruvate (+Pyruvate) and without pyruvate (None). Pyruvate concentration in the crude extracts from the cells was determined by the enzymatic and fluorometric methods described by Zhu et al. [36]. Intracellular pyruvate content was calculated as pyruvate concentration per unit dry cell weight. (**B**) DDO activity. The activity in the crude extracts is expressed as a percentage of that of the wild-type strain at None. (**C**) The *ChDDO* gene transcription is expressed as a relative ratio of that of the wild-type strain at None. (**D**) d-Asp concentration in the crude extracts from the cells of *ChDDO-*gene disrupted strain [8] was determined by the HPLC method described by Kajitani et al. [35]. Intracellular d-Asp content was calculated as d-Asp concentration per unit dry cell weight. Statistical differences were ascertained by Welch’s *t*-test: n.s., no significant; * *p* < 5 × 10^−2^; ** *p* < 5 × 10^−3^; *** *p* < 5 × 10^−4^. The values are the means of three independent experiments, and the error bars represent the standard deviations.

We hypothesized that the accumulation of pyruvate in the Δ*Chpyc1* strain affected the *ChDDO* gene expression. To verify this possibility, we measured the intracellular pyruvate content in the Δ*Chpyc1* strain. However, there was no significant difference observed between the wild-type and Δ*Chpyc1* strains (Figure 6A). To further examine the effect of pyruvate on *ChDDO* gene expression, we added 60 mM pyruvate to the culture medium and measured the intracellular pyruvate content, DDO activity, and *ChDDO* gene transcription in the strains. The intercellular pyruvate content of the wild-type and Δ*Chpyc1* strains were equal in the presence or absence of pyruvate in the medium (Figure 6A). On the other hand, DDO activity and *ChDDO* gene transcription were significantly higher in the wild-type strain but lower in the Δ*Chpyc1* strain (Figure 6B,C). We had recently reported that d-Asp uptake via amino acid permeases is involved in the inducible expression of the *ChDDO* gene [40]. Pyruvate, a monocarboxylic acid, did not inhibit the uptake of d-Asp, a dicarboxylic acid (Figure 6D). These results suggest the possibility that independent of d-Asp, the enzymatic metabolism of pyruvate by ChPyc1 might positively regulate the transcription of the *ChDDO* gene, while the enzymatic metabolism of pyruvate by other enzyme(s) might negatively regulate the transcription.

## 4. Discussion

In this study, we investigated the role of Pyc in the functional expression of *ChDDO* gene in *C. humicola*. *ChPYC1*-gene disruption resulted in retarded growth of yeast on d-Asp medium. This retardation was caused by a decreased *ChDDO* gene expression at the transcriptional level. In addition, the external addition of pyruvate significantly increased *ChDDO* gene expression in the wild-type strain but decreased the same in the Δ*Chpyc1* strain. These results suggest that *ChDDO* gene expression might be regulated by pyruvate metabolism, as well as by the presence of d-Asp. This study is the first report exploring the relationship between Pyc function and *DDO* gene expression and it might contribute to the understanding of the regulation of *DDO* gene expression in eukaryotic organisms.

In the yeast *S. cerevisiae*, *H. polymorpha*, and *P. pastoris*, and the filamentous fungus *Aspergillus nidulans*, Pyc-negative mutants require l-Asp or l-Glu to grow well on minimal media, and Pyc functions as an anaplerotic enzyme that replenishes the TCA cycle by providing oxaloacetate [23,41,42,43]. Similarly, the Δ*Chpyc1* strain showed growth retardation on a minimal medium containing glucose and NH_4_Cl as the sole carbon and nitrogen sources, respectively, whereas the growth of the Δ*Chpyc1* strain was restored by the addition of l-Asp or α-ketoglutarate (Figure 4). These results indicate that ChPyc1 can function as an anaplerotic enzyme of the TCA cycle and that, instead of ChPyc1, the Asp transaminase reaction can complement the function of Pyc by supplying oxaloacetate to the TCA cycle. On the other hand, even though ChDDO could produce oxaloacetate from d-Asp, it was not able to complement the reaction of Pyc because of the decrease in the expression of the *ChDDO* gene in the Δ*Chpyc1* strain (Figure 5). In methylotrophic yeasts, AO activity is significantly decreased or absent in *pyc1* mutants, and in the yeast *P. pastoris*, DAO activity is also significantly decreased in a *PYC1* gene disruption strain [23,27]. The decreased activity of the flavin enzymes in *pyc1* mutants is due to the lack of Pyc1-mediated FAD binding to the newly synthesized proteins in the cytosol [24,25]. On the other hand, the decrease in DDO activity in the Δ*Chpyc1* strain was caused by a decrease in the transcriptional level of the *ChDDO* gene (Figure 5B). These findings suggest that unlike AO and DAO in methylotrophic yeasts, the functional expression of the *ChDDO* gene might be regulated at the transcriptional level by the enzymatic function of ChPyc1.

The external addition of pyruvate to the culture medium enhanced *ChDDO* gene expression at the transcriptional level in the wild-type strain of *C. humicola* but repressed the same in the Δ*Chpyc1* strain (Figure 6B), even though there was no significant difference in intracellular pyruvate content between them (Figure 6A). This suggests that different pyruvate metabolism pathways might lead to different transcription of the *ChDDO* gene. Pyruvate is metabolized by pyruvate decarboxylase (Pdc), pyruvate dehydrogenase (Pdh), and additionally, by Pyc in yeasts [21]. It is possible that metabolism through Pdc and/or Pdh could decrease *ChDDO* gene transcription and that metabolism through Pyc could enhance the transcription. Pyruvate and oxaloacetate are key compounds in metabolism because they are involved in many reactions, such as carbohydrate metabolism and the biosynthesis of fatty acids and amino acids [21,44]. In *S. cerevisiae*, the transcription of *PYC1* is repressed by l-Asp and l-Glu and induced by ethanol and pyruvate [45,46,47]. In addition, Pyc1p is allosterically regulated by acetyl-CoA and l-Asp [48,49]. These findings indicate that Pyc enzyme activity is intricately regulated, depending on the nutritional status of the cell. Therefore, the decreased *ChDDO* gene expression in the absence of ChPyc1 suggests that the metabolic activity of cells might regulate gene expression. In the methylotrophic yeast *P. methanolica*, the expression of *MOD1* and *MOD2* genes encoding AO are regulated at the transcriptional level by the respiratory activity of mitochondria [50,51]. This fact suggests organelle crosstalk between mitochondria and peroxisomes through the gene expression of nucleus to maintain efficient methanol metabolism by regulating the consumption balance of oxygen between the mitochondrial respiration and the peroxisomal enzymes [51]. This regulatory mechanism seems rational based on the anaplerotic function of ChPyc1 in the TCA cycle and the molecular oxygen utilization of ChDDO in its reaction. More specifically, the decrease in oxaloacetate caused by the disruption of the *ChPYC1* gene might indicate the dysfunction of aerobic metabolism, and the accumulation of pyruvate metabolites might be involved in the regulation of *ChDDO* gene expression as a signaling factor. To further clarify the relationship between the regulation of *ChDDO* gene expression and the function of ChPyc1, it is important to investigate its physiological role and regulation of ChPyc1.

## 5. Conclusions

**Figure 7:**
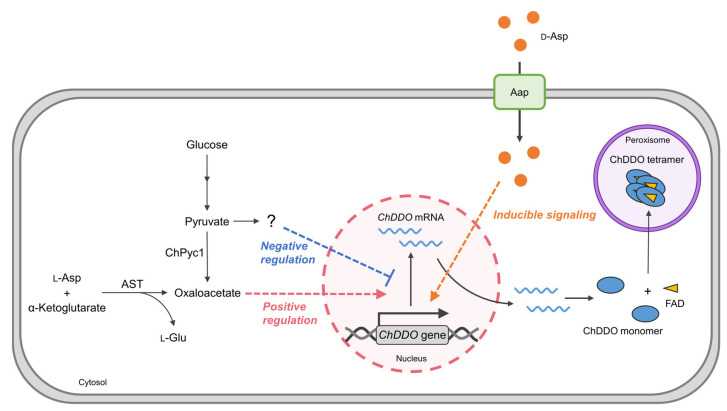
Relationship between ChPyc1 activity and *ChDDO* gene expression in the yeast *C. humicola* strain UJ1. d-Asp taken up into the cytosol by Aap induces *ChDDO* gene transcription via an unknown signaling pathway. Chpyc1 is indirectly involved in the regulation of *ChDDO* transcription via pyruvate metabolism. Pyruvate metabolisms by ChPyc1 and other enzyme(s) might regulate *ChDDO* gene inducible transcription by d-Asp positively and negatively, respectively.

In conclusion, we describe the following possible model for the relationship between the functional expression of *ChDDO* gene and ChPyc1 (Figure 7). The transcription of the *ChDDO* gene is induced via an unknown signaling pathway by d-Asp, which is taken up by amino acid permeases (Aaps). In contrast, ChPyc1 can function as an anaplerotic enzyme that supplies oxaloacetate to the TCA cycle, which can be compensated by Asp transaminase (AST) but not ChDDO. In addition, independent of the induction by d-Asp, the metabolism of pyruvate by ChPyc1 might positively regulate the transcription of the *ChDDO* gene, while that by other enzyme(s) might negatively regulate the transcription. At present, pyruvate metabolites and signaling pathways that directly regulate the transcription of the *ChDDO* gene are unknown. In future research, we aim to identify the metabolites and proteins involved in the transcription of the *ChDDO* gene to understand the whole regulatory mechanism. Information on the regulatory mechanism of *DDO* gene expression in yeast may contribute to the elucidation of the physiological functions of DDO and d-Asp in eukaryotes.

## Data Availability

The data presented in this study are available on request from the corresponding author.

## Supplementary Materials

The following are available online at https://www.mdpi.com/article/10.3390/microorganisms9122444/s1, Table S1: Primers used in this study.
