# Supplementary material for: Regulation of d-Aspartate Oxidase Gene Expression by Pyruvate Metabolism in the Yeast Cryptococcus humicola
Source: Microorganisms. 2021 Nov 27;9(12):2444. doi: 10.3390/microorganisms9122444 (PMC8708985; doi:10.3390/microorganisms9122444)
Supplement: Supplementary file 1 [file microorganisms-09-02444-s001.zip › microorganisms-1479696-supplementary.pdf]

Table S1. Primers used in this study

Primers	Sequence (5′ to 3′)
**For cDNA synthesis**	
ChPyc1F1	CGTCATGACACCCACACGTC
ChPyc1R1	TGAAGCCTAGGCCTTCTCAG
**For constructing gene-expression vector**	
pWGP3ChPyc1F	TTCGAGCTCGGTACCATGACACCCACACGTC
pWGP3ChPyc1R	GACTCTAGAGGATCCCTAGGCCTTCTCAGCCGCCTCCTC
pWGP3R	GGTACCGAGCTCGAATTC
pWGP3F	GGATCCTCTAGAGTCGAC
**For constructing gene-disrupting cassette**	
URA3F	CGAGGTCGACGGTATCG
URA3R	CAGGAAACAGCTATGAC
ChPYC1UF	GTCACCTCCCGTACAACAAG
ChPYC1UR	CGATACCGTCGACCTCGGCGATCTCGACGACCTTCTG
ChPYC1DF	GTCATAGCTGTTTCCTGCGACGGCAAGGTTGTTGACAC
ChPYC1DR	CTGGACGTCAAAGTCGGTGA
**For checking gene disruption**	
Fwd1	CAGGACTTGACCCGGTATAG
Rev1	CTCCCTCCTCCTTCTTCTTG
Fwd2	TCTCTCACTCACCACGCATC
Rev2	GTCCCAAGGCAGGTTGAATG
**For qRT-PCR**	
RTChDDOF2	CTCACGCAGACCGAGATGTG
RTChDDOR2	GTCGAGCACGCGGAAATCT
RTChTAF10F	CGAGGAGGTGACCGAGTACT
RTChTAF10R	CAGAGACAACAGGCGCTTTAGTC
